# NALCN is a potential biomarker and therapeutic target in human cancers

**DOI:** 10.3389/fgene.2023.1164707

**Published:** 2023-04-19

**Authors:** Jian He, Jie Xu, Zhiwei Chang, Jiaqin Yan, Limin Zhang, Yanru Qin

**Affiliations:** ^1^Department of Oncology, The First Affiliated Hospital of Zhengzhou University, Zhengzhou, China; ^2^ School of Public Health, Xinxiang Medical University, Xinxiang, China

**Keywords:** NALCN, pan-cancer analysis, immune infiltration, diagnosis, prognosis

## Abstract

**Background:** Sodium leak channel non-selective (NALCN), known as a voltage-independent Na^+^ channel, is increasingly considered to play vital roles in tumorigenesis and metastasis of human cancers. However, no comprehensive pan-cancer analysis of NALCN has been conducted. Our study aims to explore the potential diagnostic, prognostic and therapeutic value of NALCN in human cancers.

**Methods:** Through comprehensive application of datasets from Human Protein Atlas (HPA), The Cancer Genome Atlas (TCGA), Cancer Cell Line Encyclopedia (CCLE), Enhanced Version of Tumor Immune Estimation Resource (TIMER2.0), Tumor and Immune System Interaction Database (TISIDB), The University of Alabama at Birmingham Cancer data analysis Portal (UALCAN), cBioPortal, GeneMANIA and Search Tool for the Retrieval of Interaction Gene/Proteins (STRING) databases, we explored the potential roles of NALCN in different cancers. The differential expression, prognostic implications, pathological stages and grades, molecular and immune subtypes, diagnostic accuracy, tumor mutation burden (TMB), microsatellite instability (MSI), mismatch repair (MMR) genes, immune checkpoint genes, chemokine genes, major histocompatibility complex (MHC)-related genes, tumor-infiltrating immune cells (TIICs), promoter methylation, mutations, copy number alteration (CNA), and functional enrichment related to NALCN were analyzed.

**Results:** Most cancers lowly expressed NALCN. Upregulated NALCN expression was associated with poor or better prognosis in different cancers. Moreover, NALCN was correlated with clinicopathological features in multiple cancers. NALCN showed high diagnostic accuracy in 5 caner types. NALCN is highly linked with immune-related biomarkers, immune-related genes and TIICs. Significant methylation changes and genetic alteration of NALCN can be observed in many cancers. Enrichment analysis showed that NALCN is closely related to multiple tumor-related signaling pathways.

**Conclusion:** Our study revealed the vital involvement of NALCN in cancer. NALCN can be used as a prognostic biomarker for immune infiltration and clinical outcomes, and has potential diagnostic and therapeutic implications.

## Introduction

Most cancer patients die of metastasis process of cancers by which malignant tumor cells spread to other organs from the primary tumor (Ganesh and Massagué, 2021). Blocking tumor cell metastasis can greatly enhance the survival rate of cancer patients, while how the metastasis process is switched on remains unclear, within the complex network of tumorigenesis (Massagué and Obenauf, 2016; Dillekås et al., 2019). For a long time, NALCN was identified as a single ion channel. However, lately study has revealed that NALCN is essential for the metastasis of cancer and the transmission of normal cells (Rahrmann et al., 2022).

The NALCN protein contains 1738 amino acids and forms the integral membrane ion channel complex. NALCN is encoded by just one gene in *Homo sapiens* (Liebeskind et al., 2012; Senatore et al., 2013). NALCN has been shown to control the resting membrane potential by regulating sodium leak conductance. Moreover, NALCN has been reviewed to play a key function in excitable tissues, such as neuronal excitability, circadian and respiration rhythms (Lu et al., 2007; Chua et al., 2020; Kschonsak et al., 2020). Neurological disorders are related to NALCN gain-of-function mutations (Bend et al., 2016). In gastric and colorectal cancers, there was an enriched presence of loss-of-function mutations of NALCN. Tumor incidence was unaffected by deletion of NALCN in mouse model, but tumor cell metastasis was markedly increased (Rahrmann et al., 2022).

From the perspective of the lack of pan-cancer study and further explore the role of NALCN in cancer, we analyzed NALCN across various cancer types based on large-scale RNA-sequencing data. In the present work, we examined the expression of NALCN, the prognostic implications of NALCN, the potential clinicopathological correlations, diagnostic accuracy of NALCN, its association with immune-related markers and tumor-infiltrating immune cells (TIICs), methylation level of NALCN, mutations and copy number alteration (CNA) in NALCN, and functional enrichment analysis for NALCN by data mining analyses. The results showed that NALCN is aberrantly expressed and closely associated with clinicopathological features in multiple cancers. In some cancers, upregulated expression of NALCN is detrimental to survival, while in others, it is beneficial. The receiver operating characteristic (ROC) tests show high diagnostic accuracy of NALCN. These results suggested that NALCN has an important impact on the prognosis of cancer patients and present promising diagnostic value for cancer, while its role varies according to the type of cancer. Moreover, NALCN is highly linked with immune-related biomarker, immune-related genes and TIICs. Significant methylation changes and genetic alteration of NALCN can be observed in many cancers. Enrichment analysis found that NALCN is closely related to multiple tumor-related signaling pathways. Getting these data together, the results suggest NALCN is a prognostic marker of immune infiltration, as well as clinical outcomes, and has potential diagnostic and therapeutic implications.

## Materials and methods

### NALCN expression analysis in pan-cancer

The Human Protein Atlas (HPA) (http://www.proteinatlas.org/) database was used to estimate the mRNA expression levels of NALCN in normal tissues (Uhlén et al., 2015). Pan-cancer sequencing data of NALCN from The Cancer Genome Atlas (TCGA) (https://www.cancer.gov/about-nci/organization/ccg/research/structural-genomics/tcga) were collected for analysis through their portal websites (Tomczak et al., 2015). Cancer Cell Line Encyclopedia (CCLE) (https://sites.broadinstitute.org/ccle) database was applied to obtain mRNA expression levels of NALCN in cell lines (Nusinow et al., 2020). We further investigated NALCN expression in 33 types of cancer and normal tissues from TCGA datasets using the enhanced version of tumor immune estimation resource (TIMER2.0) (http://timer.cistrome.org/) (Li et al., 2020). The “Gene_DE” module was explored with input of “NALCN.” Log2 transformation was conducted on the expression data. R software and “ggplot2” R package were applied for analysis and visualization.

### Immunohistochemistry staining of NALCN

Using the HPA database, Immunohistochemistry (IHC) images of tumor tissues were compared with the corresponding IHC images of normal tissues, to analyze the differential expression of NALCN protein.

### Prognostic potential analysis of NALCN in pan-cancer

In each TCGA cancer type, we conducted Cox proportional hazards regression models and Kaplan-Meier (KM) analysis to investigate how NALCN expression correlates with patient’s overall survival (OS), progression free interval (PFI) and disease specific survival (DSS). The “forestplot” and “survival” R packages were employed.

### Correlation analysis of NALCN and clinicopathological characteristics

We used the Tumor and Immune System Interaction Database (TISIDB) (http://cis.hku.hk/TISIDB/index.php) to explore the correlation between NALCN and pathological stages, as well as histological grades of cancers. Using the TISIDB database, we further evaluated the relationships between the expression of NALCN and molecular subtypes or immune subtypes in pan-cancer (Ru et al., 2019).

### Analysis of the diagnostic value of NALCN

To evaluate the diagnostic value of NALCN, the ROC curve analysis was conducted by the “pROC” R package. The ROC curves were made using the “ggplot2” R package. The area under the curve (AUC) more than 0.8 represent high diagnostic accuracy.

### Analysis of NALCN expression and immune-related biomarker

Based on the TCGA data, Spearman’s coefficient was used to evaluate associations between the expression level of NALCN and tumor mutation burden (TMB) or microsatellite instability (MSI) in different cancer types. Using “ggradar” and “ggplot2” R packages, radar plots were displayed as the final results. Furthermore, the correlation between NALCN expression and MMR genes, immune checkpoint genes, chemokine genes, major histocompatibility complex (MHC)-related genes in various TCGA cancer types was investigated using Spearman’s correlation method. The results were exhibited as heatmaps using “ggplot2” R package.

### Immune infiltration analysis of NALCN

We used the “estimate” R package to estimate tumor purity in 33 human cancers. Concretely, it was calculated based on the immune score and stromal score, which respectively represent the infiltration of immune cells and the stromal components in tumor tissue. ESTIMATE score reflects both components integrated and indirectly indicate tumor purity. Correlations between NALCN expression and these three kinds of score in the top 3 cancers were shown as scatter plots.

### NALCN methylation profile in pan-cancer

In this study, the promoter methylation level of NALCN in different cancers was examined using The University of Alabama at Birmingham Cancer data analysis Portal (UALCAN) (http://ualcan.path.uab.edu/). Moreover, the association between NALCN promoter methylation level and tumor stage, as well as nodal metastasis status was investigated through UALCAN database.

### Genetic alteration analysis of NALCN

Using the cBioPortal database (http://www.cbioportal.org/), we investigated the genetic alterations of NALCN in TCGA pan-cancer datasets. The “pan-cancer analysis of whole genomes (ICGC/TCGA, Nature 2020)" dataset, “Oncoprint,” “Cancer Type Summary,” “Plots,” “Mutations,” and “Comparison/Survival” modules were used to investigate the mutation landscape and CNA of NALCN in different cancers.

### Gene-related enrichment analysis

GeneMANIA database (http://genemania.org/) was used to obtain NALCN gene-gene interaction network. We input NALCN (protein name) and *H. sapiens* (organism) to query the Search Tool for the Retrieval of Interaction Gene/Proteins (STRING) database (https://string-db.org/). Then, the basic parameters were set as: No more than 50 interactors (maximum number of interactors to show), medium confidence 0.400 (minimum required interaction score) and evidence (the meaning of network edges). Finally, the enrichment analysis was performed with “Cluster Profiler” R package, while enrichment pathways were visualized with “ggplot2” R package.

### Statistical analysis

In the present work, R software (version 4.2.1) was used for the analysis. Wilcoxon’s test were used for the comparison between two groups. The correlation between NALCN expression and interest targets was assessed by Spearman correlation test. Cox proportional hazards regression models were used to calculate the Hazard Ratio (HR). KM analysis and log-rank test were performed to analyze the survival outcome. *p*-value less than 0.05 were considered statistically significant (*p < 0.05, **p < 0.01, ***p < 0.001).

## Results

### NALCN expression levels in normal and cancer tissues

**FIGURE 1:**
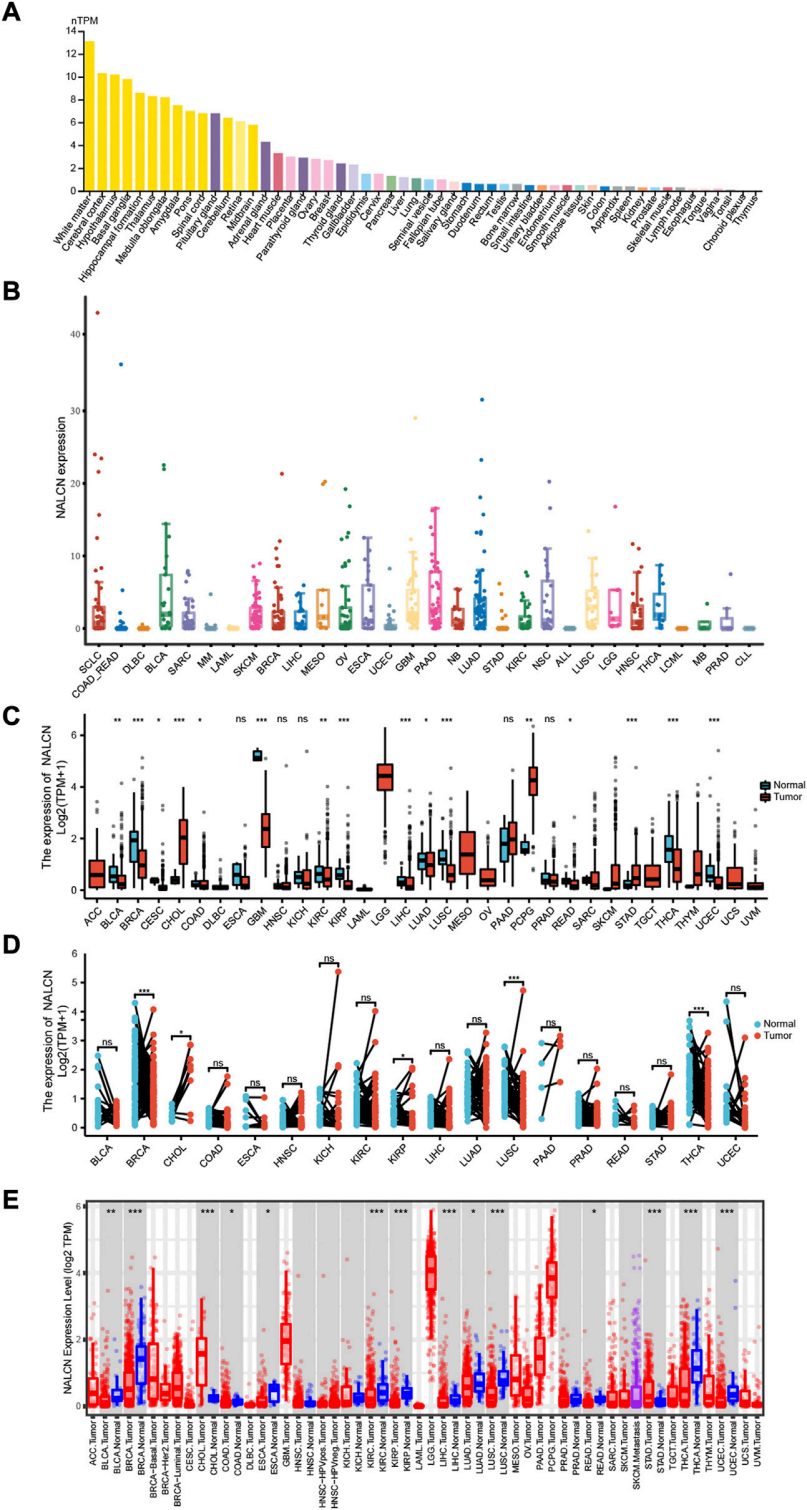
Differential expression of NALCN. **(A)** Expression of NALCN in normal tissues. **(B)** Expression of NALCN in cancer cell lines. **(C)** Comparison of NALCN between tumor and normal tissues. **(D)** Comparison of NALCN between paired tumor and normal tissues. **(E)** Comparison of NALCN between tumor and normal tissues in TIMER 2.0. *p < 0.05, **p < 0.01, ***p < 0.001, ns *p* ≥ 0.05.

Human normal tissues were examined using the HPA database for NALCN mRNA expression under physiological conditions, based on HPA and GTEx datasets. NALCN was expressed almost in all normal tissues, with the exception of the choroid plexus and thymus. The expression of NALCN was tissue specific. Compared with other organs, enhanced NALCN expression was detected in brain. The mRNA expression level of NALCN was highest in white matter (Figure 1A). Moreover, we examined NALCN mRNA expression in CCLE cancer cell lines (Figure 1B). Taking TCGA data, compared with adjacent normal tissues, the NALCN expression level were significantly downregulated in bladder urothelial carcinoma (BLCA), breast invasive carcinoma (BRCA), cervical squamous cell carcinoma and endocervical adenocarcinoma (CESC), colon adenocarcinoma (COAD), glioblastoma multiforme (GBM), kidney renal clear cell carcinoma (KIRC), kidney renal papillary cell carcinoma (KIRP), liver hepatocellular carcinoma (LIHC), lung adenocarcinoma (LUAD), lung squamous cell carcinoma (LUSC), rectum adenocarcinoma (READ), thyroid carcinoma (THCA), uterine corpus endometrial carcinoma (UCEC), but upregulated in cholangiocarcinoma (CHOL), pheochromocytoma and paraganglioma (PCPG), stomach adenocarcinoma (STAD) (Figure 1C). Furthermore, using the TCGA datasets, we estimated NALCN expression in paired cancer and normal tissues. NALCN was significantly lower in BRCA, KIRP, LUSC, THCA, while higher in CHOL, than in paired normal tissues (Figure 1D). Besides, we compared the differential expression of NALCN mRNA in human pan-cancer using the TIMER2.0 database and found it was consistent with the above analysis (Figure 1E). These results indicate that the expression of NALCN was aberrant in multiple cancer types which suggest that NALCN could be a crucial tool for cancer diagnosis.

### NALCN protein expression in pan-cancer

**FIGURE 2:**
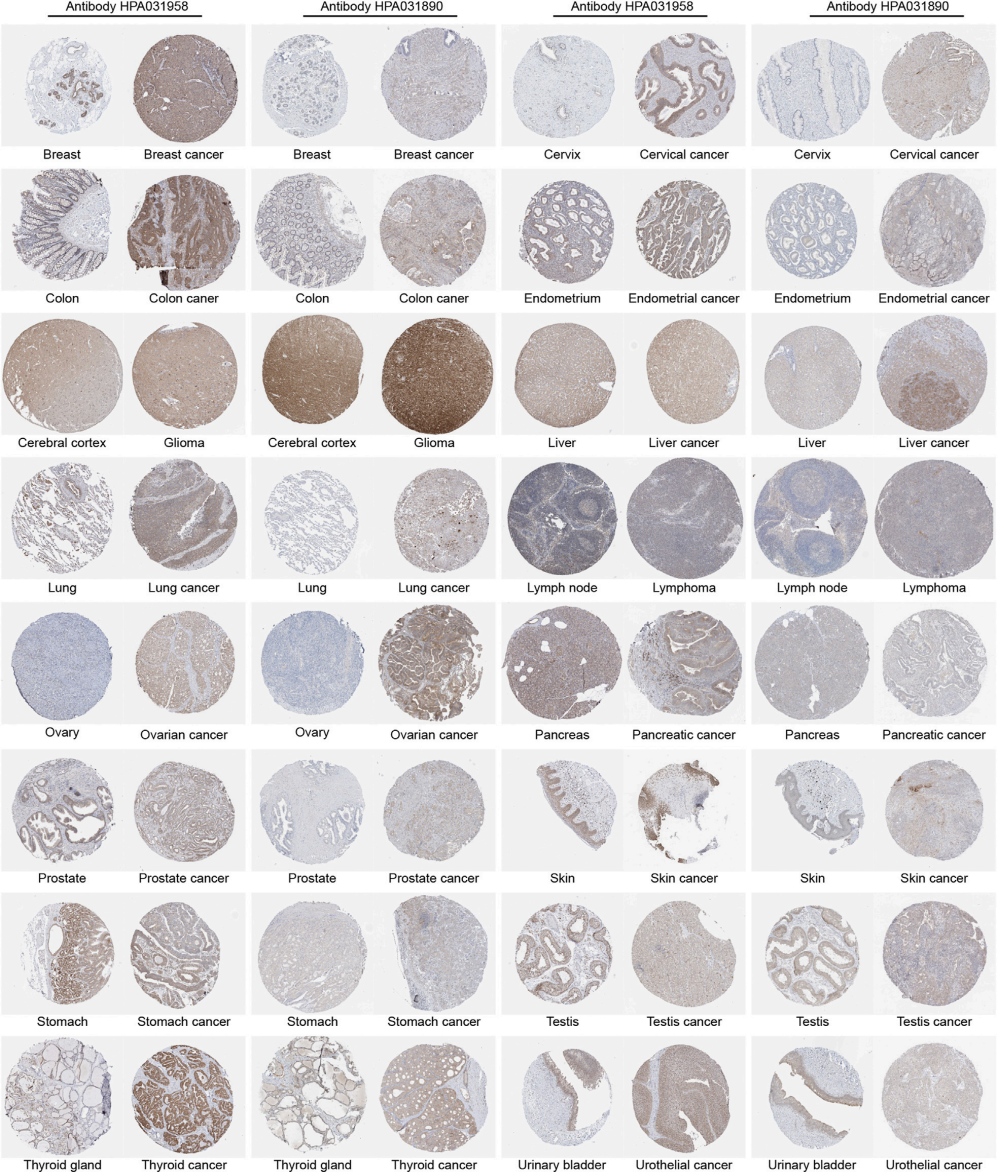
Representative immunohistochemical images of NALCN protein expression level in normal and tumor tissues (antibodies for immunohistochemistry: HPA031958, HPA031890).

IHC results showed NALCN protein localizing in the cytoplasm and membrane. The expression of NALCN protein was higher in breast, cervical, colon, endometrial, liver, lung, ovarian, prostate, skin, and thyroid cancer than in normal tissues (Figure 2). We noted that IHC staining for NALCN was performed using antibodies with different clones might produce variable results. There was low consistency between IHC staining and RNA expression data of NALCN.

### Prognostic value of NALCN in pan-cancer

**FIGURE 3:**
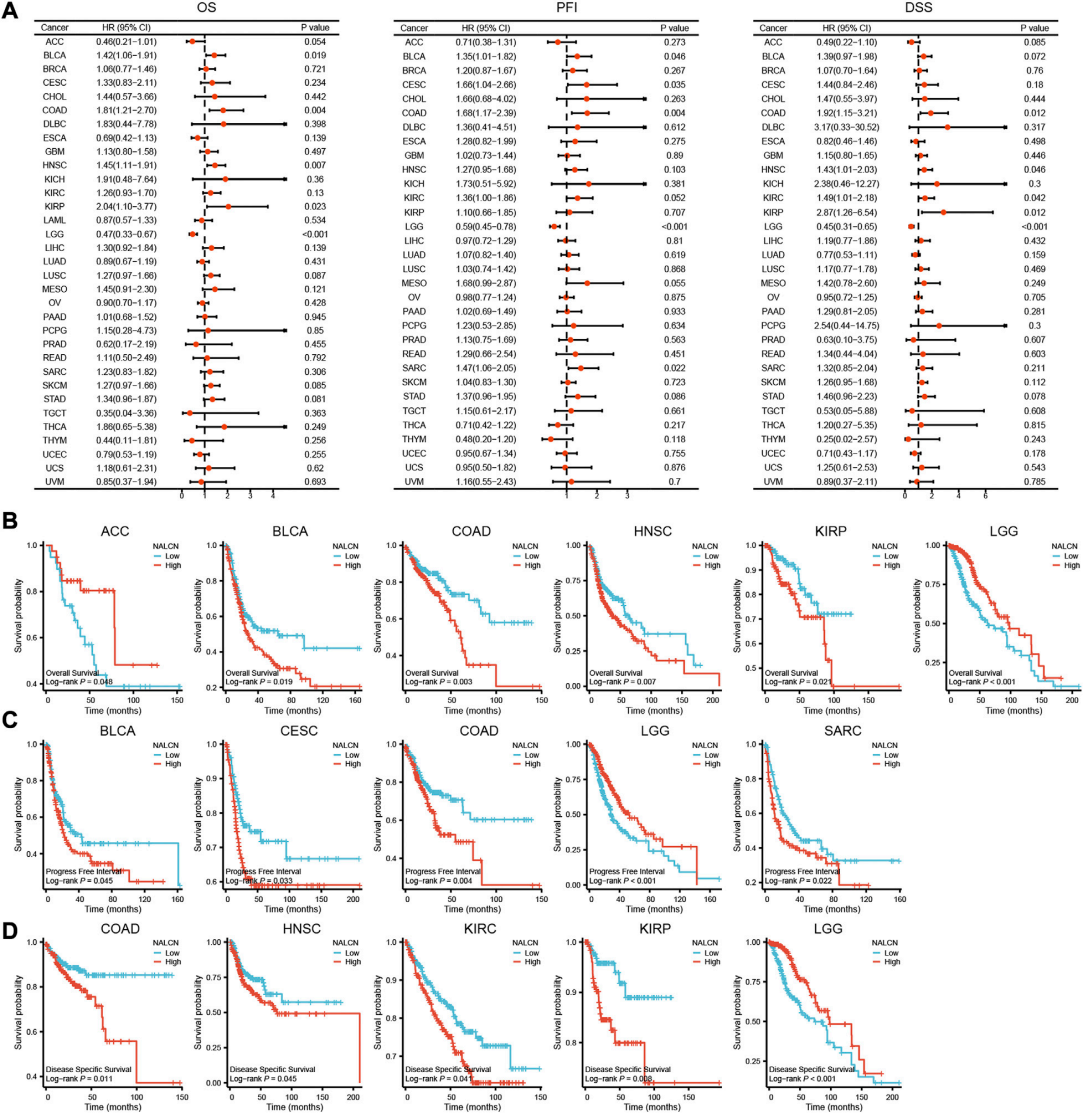
Correlation between NALCN expression and survival prognosis. **(A)** Forest plot of OS, PFI and DSS correlations in TCGA. **(B)** Kaplan-Meier analysis of the correlation between NALCN and OS. **(C)** Kaplan-Meier analysis of the correlation between NALCN and PFI. **(D)** Kaplan-Meier analysis of the correlation between NALCN and DSS.

Using data from the TCGA database, results from 33 types of cancer in Cox regression illustrated that NALCN expression level was correlated with OS in adrenocortical carcinoma (ACC), BLCA, COAD, head and neck squamous cell carcinoma (HNSC), KIRP and brain lower grade glioma (LGG). NALCN was a risk factor for BLCA, COAD, HNSC and KIRP, while it was a protective factor for ACC and LGG (Figure 3A). KM survival analysis indicated that higher NALCN expression was associated with a poorer OS in BLCA, COAD, HNSC, and KIRP, while with better OS in ACC and LGG (Figure 3B). For PFI, increased NALCN was a high-risk factor for BLCA, CESC, COAD, and sarcoma (SARC), and was a low-risk factor for LGG (Figure 3A). The results of KM curves for PFI indicated that high expression of NALCN was correlated with a worse PFI in BLCA, CESC, COAD, and SARC, while low expression of NALCN was correlated with poorer PFI in LGG (Figure 3C). Moreover, NALCN exhibited a significant prognostic value in COAD, HNSC, KIRC, KIRP, and LGG through Cox regression analysis for DSS (Figure 3A). KM of DSS analysis demonstrated that high NALCN expression had shortened DSS in patients with COAD, HNSC, KIRC, and KIRP, however had lengthened DSS in patients with LGG (Figure 3D).

### Correlation analysis between NALCN expression and clinicopathological features

**FIGURE 4:**
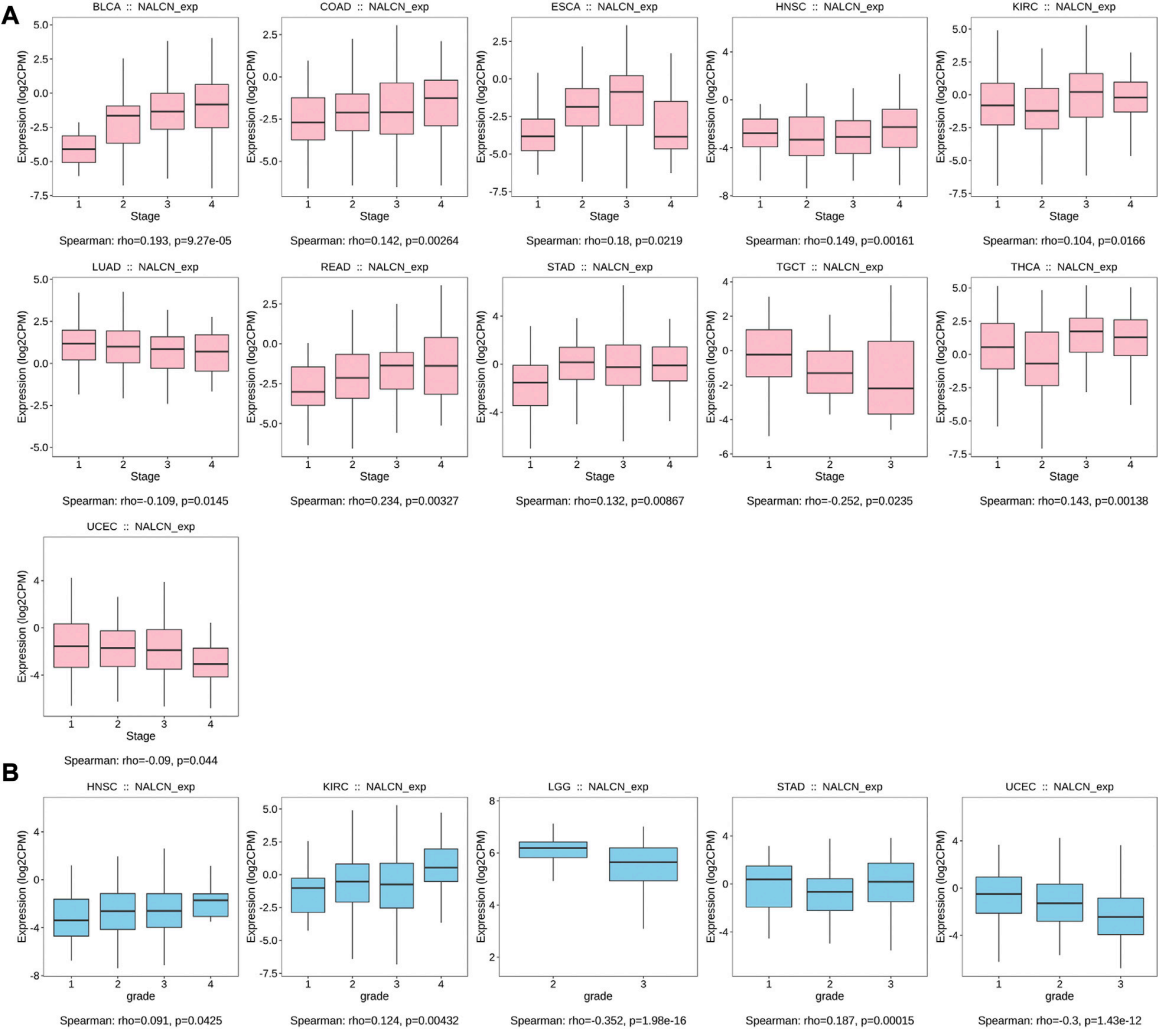
Correlation of NALCN expression and clinicopathological parameters (stage, grade) across different cancer types. **(A)** Correlation of NALCN with stages of BLCA, COAD, ESCA, HNSC, KIRC, LUAD, READ, STAD, TGCT, THCA, UCEC, based on the TISIDB database. **(B)** Correlation of NALCN with grades of HNSC, KIRC, LGG, STAD, UCEC, based on the TISIDB database.

**FIGURE 5:**
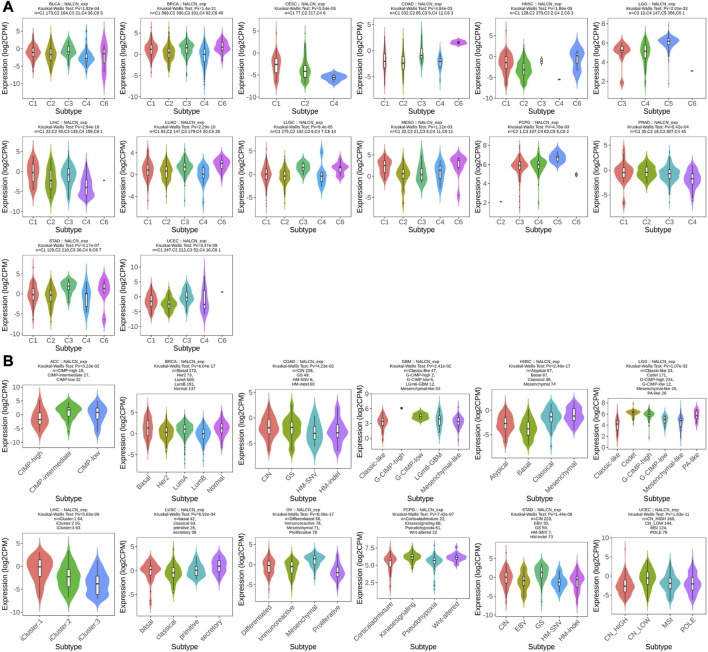
Correlation of NALCN expression and clinicopathological parameters (immune subtype, molecular subtype) across different cancer types. **(A)** Correlation of NALCN with immune subtypes (C1: wound healing, C2: IFN-gamma dominant, C3: inflammatory, C4: lymphocyte depleted, C5: immunologically quiet, C6: TGF-b dominant) of BLCA, BRCA, CESC, COAD, HNSC, LGG, LIHC, LUAD, LUSC, MESO, PCPG, PRAD, STAD, UCEC, based on the TISIDB database. **(B)** Correlation of NALCN with molecular subtypes of ACC, BRCA, COAD, GBM, HNSC, LGG, LIHC, LUSC, OV, PCPG, STAD, UCEC, based on the TISIDB database.

We examined the relationship between NALCN mRNA expression level and patient’s clinicopathological features in pan-cancer using TISIDB tool. The results showed that the expression of NALCN was significantly associated with tumor stage in BLCA, COAD, esophageal carcinoma (ESCA), HNSC, KIRC, LUAD, READ, STAD, testicular germ cell tumors (TGCT), THCA, and UCEC (Figure 4A). With elevated NALCN expression, higher histological tissue grades were shown in HNSC, KIRC, and STAD, while in LGG and UCEC reversely (Figure 4B). Moreover, the relationship between NALCN expression and immune or molecular subtypes in pan-cancer was investigated. The results revealed that the expression of NALCN was correlated with 6 immune subtypes in 14 cancer types, including BLCA, BRCA, CESC, COAD, HNSC, LGG, LIHC, LUAD, LUSC, mesothelioma (MESO), PCPG, prostate adenocarcinoma (PRAD), STAD, and UCEC (Figure 5A). Meanwhile, a significant correlation between NALCN and different molecular subtypes existed in 12 cancer types, including ACC, BRCA, COAD, GBM, HNSC, LGG, LIHC, LUSC, ovarian serous cystadenocarcinoma (OV), PCPG, STAD, and UCEC (Figure 5B).

### Diagnostic value of NALCN for cancers

**FIGURE 6:**
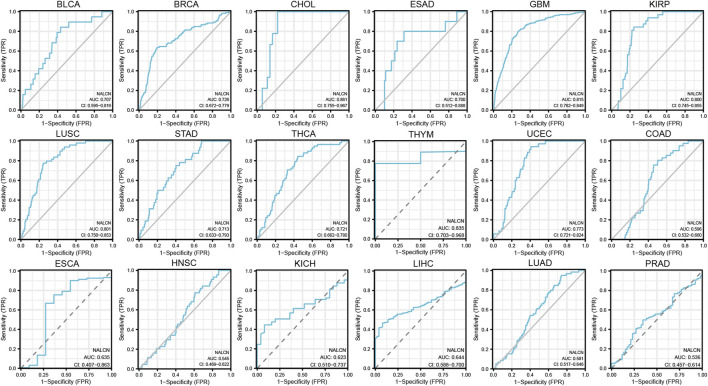
AUC of ROC curves showed the diagnosis performance of NALCN in the TCGA cohorts.

Diagnostic accuracy of NALCN was evaluated with ROC curves. The AUC of ROC analysis shows that in 5 cancer types, the test has high diagnostic accuracy (AUC ≥ 0.8), including CHOL, GBM, KIRP, LUSC and thymoma (THYM); in 6 cancer types, it has relative diagnostic accuracy (0.7–0.8), including BLCA, BRCA, esophagus adenocarcinoma (ESAD), STAD, THCA and UCEC; and in 7 cancer types, it has low diagnostic accuracy (0.5–0.7), including COAD, ESCA, HNSC, kidney chromophobe (KICH), LIHC, LUAD and PRAD (Figure 6). AUC more than 0.8 represent excellent discrimination. Consequently, these results suggested that NALCN may be a promising diagnostic biomarker for cancers.

### Correlation between NALCN and immune-related biomarker

**FIGURE 7:**
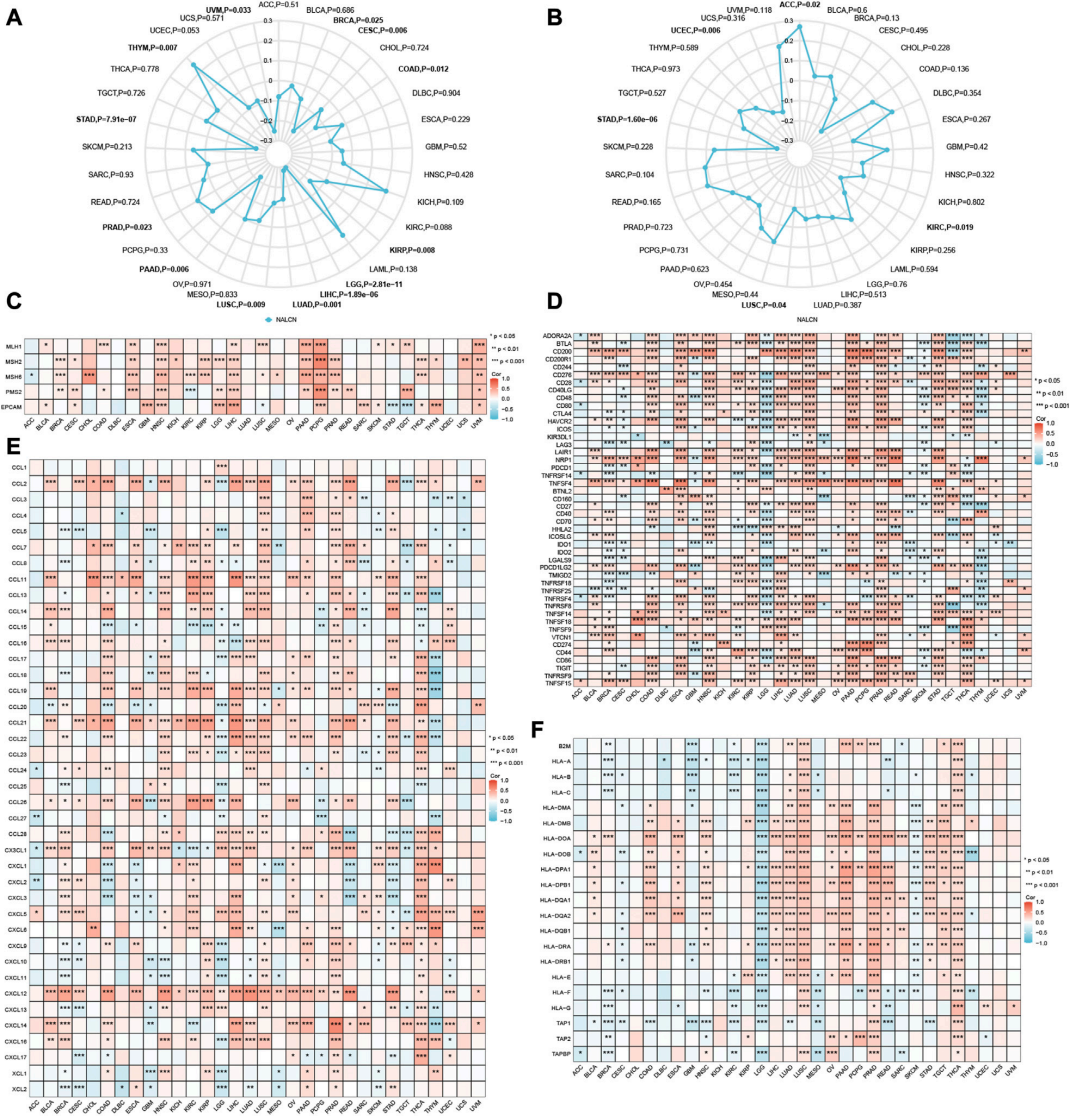
Correlations of NALCN expression and immune-related biomarker. **(A)** Radar map of correlation between NALCN expression and TMB. The curve in blue reveals the correlation coefficient. **(B)** Radar map of correlation between NALCN expression and MSI. The curve in blue reveals the correlation coefficient. **(C)** Heatmap of correlation between NALCN expression and MMR genes. **(D)** Heatmap of correlation between NALCN expression and immune checkpoint genes. **(E)** Heatmap of correlation between NALCN expression and chemokine genes. **(F)** Heatmap of the correlation between NALCN expression and MHC-related genes. *p < 0.05, **p < 0.01, ***p < 0.001.

Both TMB and MSI are mutation biomarkers that relate to the response of immunotherapy. We investigated the relationship between the expression of NALCN and either TMB or MSI in all TCGA tumors. The expression of NALCN was positively connected with TMB in PRAD and THYM, whereas negatively connected with TMB in BRCA, CESC, COAD, KIRP, LGG, LIHC, LUAD, LUSC, pancreatic adenocarcinoma (PAAD), STAD and uveal melanoma (UVM) (Figure 7A). Additionally, the expression of NALCN is negatively related to MSI in KIRC, LUSC, STAD, and UCEC, and positively related to ACC (Figure 7B). As a result of the discovery that NALCN expression is associated with TMB and MSI, more investigation about the relationship between the expression of NALCN and carcinogenesis was necessary, specifically a correlation to MMR deficiencies. Here, we investigated the correlation between NALCN expression and putative MMR genes, comprising MLH1, MSH2, MSH6, PMS2, and EPCAM. As a consequence, there was a strong correlation between NALCN expression and MMR genes in 32 cancers, aside from lymphoid neoplasm diffuse large B-cell lymphoma (DLBC), LUAD, OV, and UCEC. Especially, MLH1, MSH2, MSH6, PMS2, and EPCAM are all positively correlated with NALCN in HNSC, LIHC, PCPG, and UVM (Figure 7C). These results suggest that NALCN expression in cancer is highly correlated with carcinogenesis and immune checkpoint blockade (ICB) response.

### Correlation between NALCN and immune-related genes

In this work, the correlation between NALCN expression and immune checkpoint genes was analysed. Notably, there was a significant correlation between NALCN and many immune checkpoint genes in most cancers, such as BLCA, COAD, ESCA, KIRP, LIHC, LUSC, OV, PAAD, PRAD, READ, and THCA, and so forth. (Figure 7D). It's important to note that the expression of NALCN was negatively associated with immune checkpoint genes in LGG and GBM. NALCN expression was positively associated with majority of chemokine genes in BLCA, CHOL, HNSC, KIRC, KIRP, LIHC, LUAD, LUSC, OV, PAAD, PRAD, THCA, and UVM, and negatively associated with chemokine genes in LGG (Figure 7E). Additionally, we can found NALCN was positively correlated with MHC-related genes in LIHC, LUSC, OV, PAAD, PRAD, TGCT, and THCA, while negatively correlated with MHC-related genes in ACC, CESC, GBM, KIRC, LGG, MESO, skin cutaneous melanoma (SKCM) (Figure 7F). These findings demonstrated that a possible synergy between NALCN and known immune-related genes for regulation of tumor immune response.

### Association between NALCN expression and immune infiltration in pan-cancer

**FIGURE 8:**
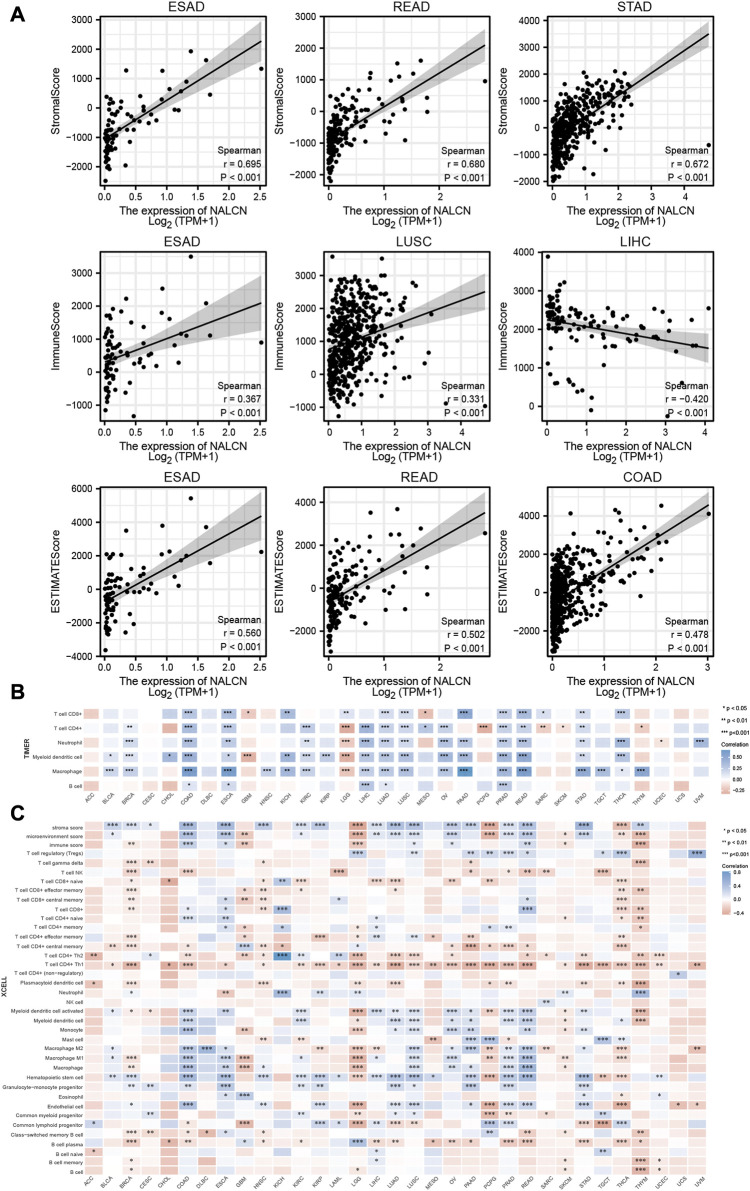
Relationship between NALCN expression and immune cell infiltration. **(A)** Top three scatter plots of correlation between NALCN and StromalScore, Immune Score, ESTIMATE Score in different cancers. **(B)** The correlations of NALCN and immune cell infiltration in cancers based on TIMER algorithms. **(C)** The correlations of NALCN and immune cell infiltration in cancers based on XCELL algorithms.

Initially, we examined the relationship between the expression of NALCN and tumor purity to determine whether NALCN plays a role in immune infiltration in pan-cancer, through stromal score, immune score and ESTIMATE score. The results showed that NALCN expression significantly related to immune and stromal scores in most cancer types. Top 3 cancers most significantly related to NALCN expression were ESAD, READ and STAD (stromal score); ESAD, LUSC and THYM (immune score); ESAD, READ and COAD (ESTIMATE score) (Figure 8A). TIICs have a strong relationship with the development and metastasis of malignant tumors. To further investigate whether NALCN has an effect on the tumor immune microenvironment, we analyzed the correlation between NALCN expression and the level of TIICs from different TCGA cohort tumors, according to TIMER and XCELL algorithms. It was found that the expression of NALCN was significantly associated with CD8^+^ T cells infiltration level among 14 cancer types, CD4^+^ T cells among 17 cancer types, neutrophil among 16 cancer types, DCs among 19 cancer types, macrophages among 19 cancer types, and B cells among 5 cancer types (Figure 8B). In COAD, ESCA, LUAD, and PRAD, there was positive correlation between NALCN and all these 6 types immune cells (Figure 8B). The relationship between NALCN expression and 38 subtypes of TIICs was further confirmed by XCELL algorithm. The result implied that NALCN expression had a significant relation with TIICs for most types of cancer. Especially, the expression of NALCN was negatively associated with most TIICs levels in BRCA, LGG, SKCM, PCPG, THCA, and THYM, and positively associated with most TIICs in COAD, ESCA, LUSC, PRAD, and READ (Figure 8C). Therefore, it indicated that NALCN expression was strongly related to the degree of TIICs in cancer.

### NALCN methylation profile in pan-cancer

**FIGURE 9:**
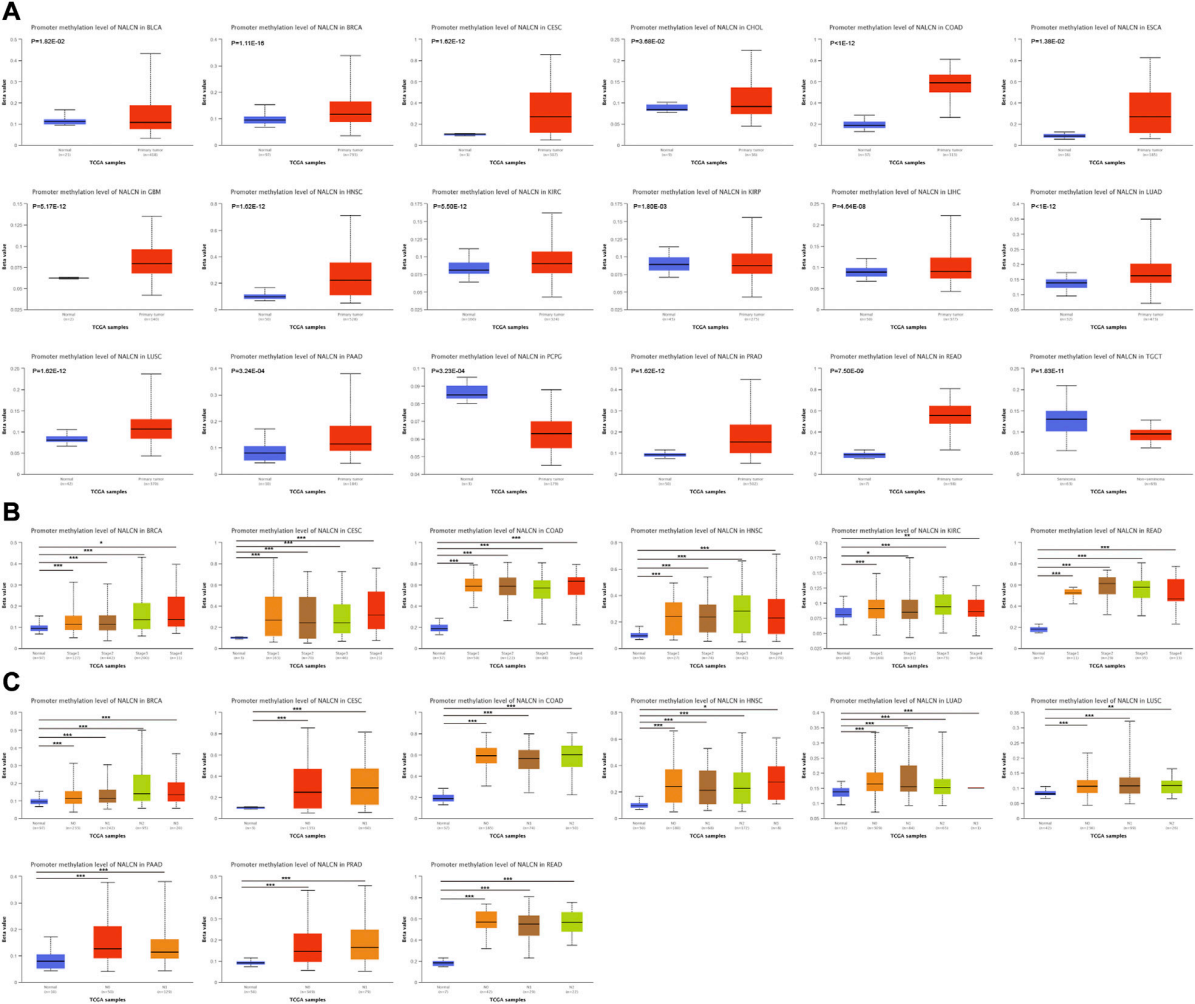
The promoter methylation level of NALCN in cancers. **(A)** The promoter methylation level of NALCN between tumor and normal samples. **(B)** The promoter methylation level of NALCN between tumor with different stage and normal samples. **(C)** The promoter methylation level of NALCN between tumor with different lymph node metastasis status and normal samples. *p < 0.05, **p < 0.01, ***p < 0.001.

Methylation level of NALCN promoter in tumor and normal tissues was evaluated by UALCAN portal. We found that the promoter methylation level of NALCN were significantly higher in BRCA, CESC, CHOL, COAD, ESCA, GBM, HNSC, KIRC, LUAD, LUSC, PAAD, PRAD, and READ, but lower in PCPG, compared to normal tissues (Figure 9A). It was also observed that the methylation level of NALCN promoter was increased in each stage tumor tissues of BRCA, CESC, COAD, HNSC, KIRC, and READ (Figure 9B), and any lymph node metastasis status of BRCA, CESC, COAD, HNSC, LUAD, LUSC, PAAD, PRAD, and READ, than those in normal tissues (Figure 9C). The above findings indicate that the promoter methylation of NALCN has a negative relation with its mRNA expression and NALCN might be an anti-oncogene in cancers.

### Genetic alteration of NALCN in pan-cancer

**FIGURE 10:**
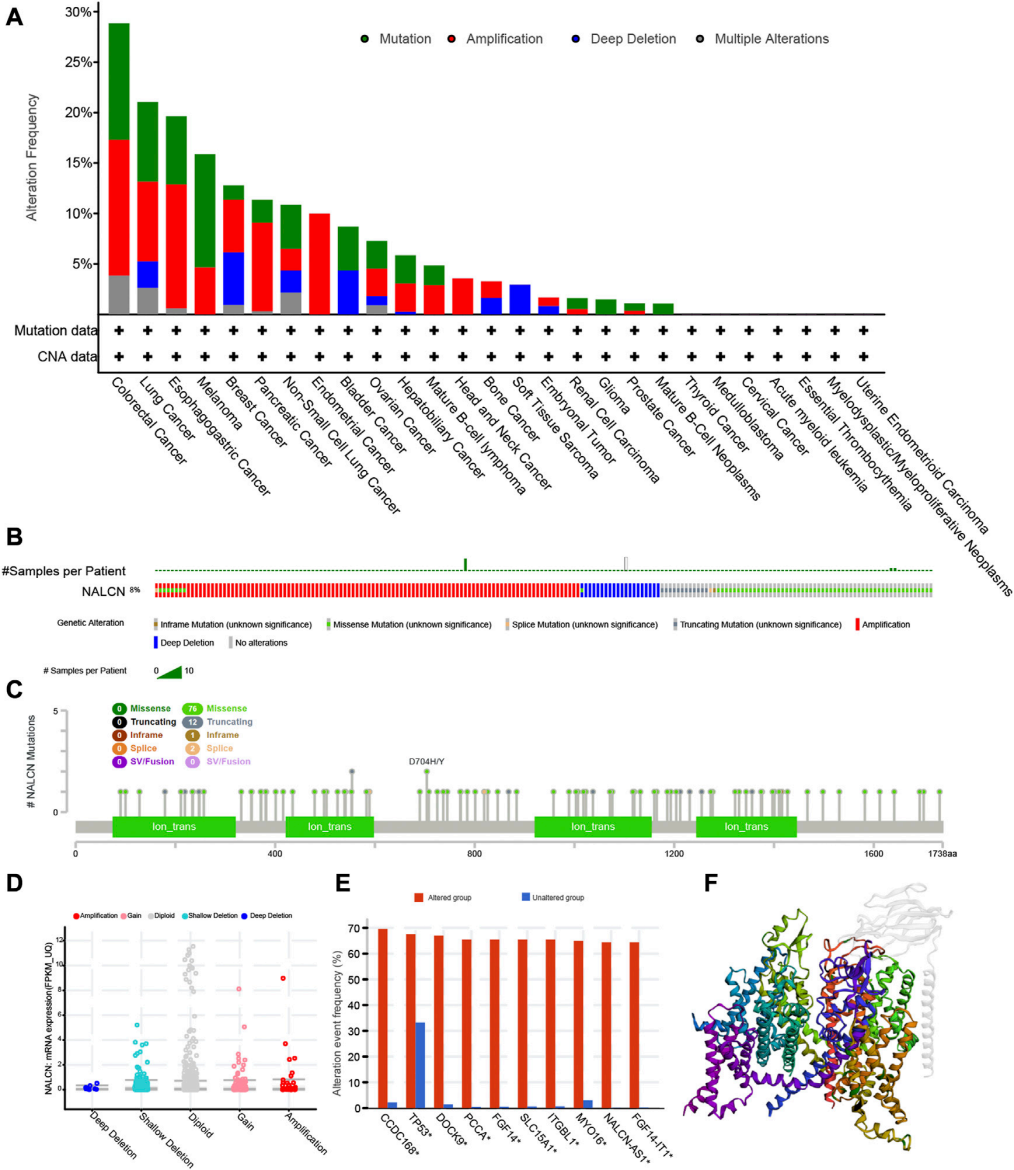
The genetic alterations of NALCN in different tumors of TCGA. **(A)** Alterations summary of NALCN for the TCGA tumors. **(B)** Summary of NALCN structural variant, mutations, and copy-number alterations. **(C)** The mutation types, number, and sites of the NALCN genetic alterations. **(D)** The alteration types of NALCN in pan-cancer. **(E)** The alteration frequency of related genes in NALCN altered group and unaltered group. **(F)** The 3D structure of NALCN protein.

From the TCGA cohorts, NALCN genetic alteration status was explored in various tumor samples by the cBioPortal database. It was found that colorectal cancer (CRC) displayed the highest frequency of NALCN gene alterations, with mutation as the primary type. Another major type of gene alterations was “amplification” of CNA in CRC, with an alteration frequency of 13.46%. NALCN amplification is observed in all cases of endometrial cancer and head and neck cancer with genetic alteration (Figure 10A). Genetic alterations of NALCN typically occur in three forms: amplification, missense mutation, and deep deletion (Figure 10B). Figure 10C further demonstrated genetic alterations in NALCN with regard to the types, sites and case numbers. Missense mutation was the major alteration type, whereas D704H/Y changes were found in 83 cases of CRC and 200 cases of STAD (Supplementary Table S1). The most frequent CNA in NALCN was diploid, shallow deletion and gain (Figure 10D). Compared with the unaltered group, the gene alteration of CCDC168, TP53, DOCK9, PCCA, FGF14, SLC15A1, ITGBL1, MYO16, NALCN-AS1, and FGF14-IT1 were more common in the NALCN altered group (Figure 10E). Figure 10F illustrates the 3D structure of NALCN protein.

### Enrichment analysis of NALCN-related partners

**FIGURE 11:**
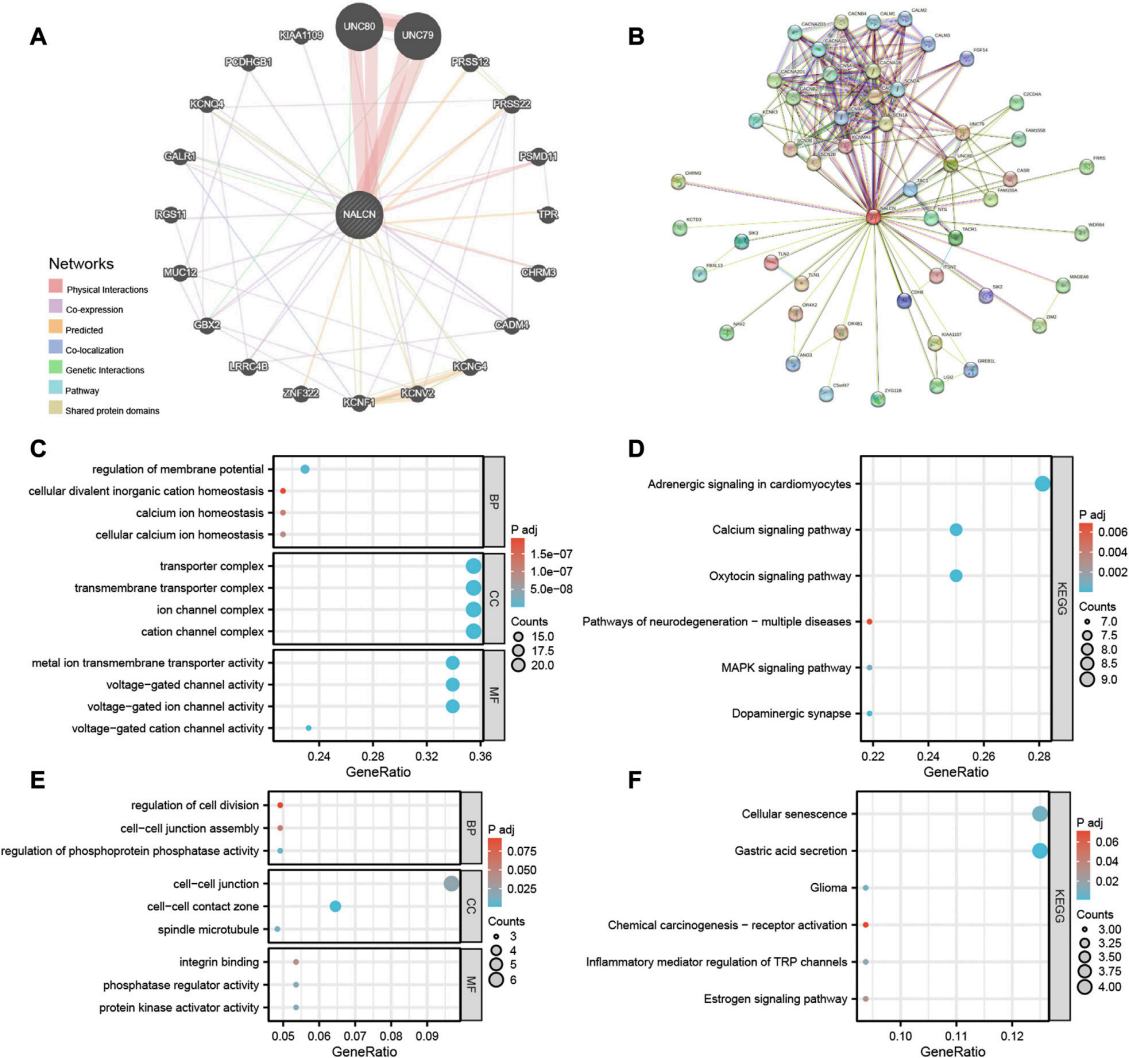
Gene-gene interaction network and enrichment analysis. **(A)** Gene-gene interaction network of NALCN from GeneMANIA. **(B)** Protein network of NALCN-binding proteins generated using STRING. **(C)** GO analysis, including the biological processes (BP), cellular components (CC), molecular functions (MF). **(D)** KEGG pathway analysis. **(E)** Representative GO analysis related to tumor. **(F)** Representative KEGG pathway analysis related to tumor.

The gene-gene interaction network of NALCN was obtained using GeneMANIA. According to the result, the 20 genes most closely associated with NALCN were identified, in which NALCN significant physical interactions with UNC80, UNC79, PSMD11 and CHRM3 (Figure 11A). Moreover, the functional analysis demonstrated that NALCN was prominently associated with voltage-gated cation channel activity, transmembrane transporter complex and potassium channel activity. The most relevant 50 NALCN-binding proteins were obtained by the STRING database. Interaction network of these proteins is exhibited in Figure 11B. UNC79, UNC80, and CHRM3 were common members from the intersection analysis of the above two groups. Enrichment analysis was conducted based on integrating the two sets of data (Supplementary Table S2). Regarding the GO terms, the biological process (BP) was mainly enriched in regulation of membrane potential, cellular divalent inorganic cation homeostasis, calcium ion homeostasis. The cellular component (CC) was primarily involved in transporter complex, transmembrane transporter complex, ion channel complex. The primary molecular function (MF) contained metal ion transmembrane transporter activity, voltage-gated channel activity, voltage-gated ion channel activity (Figure 11C). Top 3 KEGG enrichment pathways were adrenergic signaling in cardiomyocytes, oxytocin signaling pathway, calcium signaling pathway (Figure 11D). We also found that BP enriched in regulation of phosphoprotein phosphatase activity, cell-cell junction assembly, regulation of cell division; CC enriched in spindle microtubule, protein kinase activator activity, phosphatase regulator activity; MF enriched in protein kinase activator activity, phosphatase regulator activity, integrin binding (Figure 11E); KEGG pathway enriched in cellular senescence, gastric acid secretion, glioma, chemical carcinogenesis-receptor activation, inflammatory mediator regulation of TRP channels and estrogen signaling pathway (Figure 11F). These results suggested that NALCN is closely related to tumor-related signaling pathways.

## Discussion

Metastasis can occur many years after local cancer resection, or even without the presence of a primary tumor (Pantel et al., 2008; Kolling et al., 2019). Some genes promote metastasis, including several ion channels that regulate gene transcription by affecting transmembrane voltage and causing a metastasis-like phenotype (House et al., 2010; Wang et al., 2020; Sheth and Esfandiari, 2022). NALCN is a voltage-independent Na^+^ channel and is located in the plasma membrane, which belongs to the superfamily of four-domain ion channels (Lee et al., 1999). NALCN plays a crucial role in maintaining the resting and excitable membrane potentials of cells, and is involved in multiple processes, for instance, sensitivity to volatile anesthetics and locomotive behavior (Lu et al., 2007; Ren, 2011; Cochet-Bissuel et al., 2014). In addition, NALCN gene may be a susceptibility locus for a variety of diseases, including alcoholism, alzheimer’s disease, autism, bipolar disorder, cardiac disease, epilepsy and schizophrenia (Cochet-Bissuel et al., 2014). Studies reveal that NALCN is expressed in some cancers, such as glioblastoma, non-small cell lung cancer (NSCLC), pancreatic cancer, small cell lung cancer (SCLC) and tumor-derived endothelial cells (Lee et al., 2013; Cochet-Bissuel et al., 2014; Djamgoz, 2020). NALCN was a key gene in the malignant transformation of human normal liver cell lines (Chen et al., 2018). Genetic association studies revealed that single nucleotide polymorphism (SNP) of NALCN gene was associated with NSCLC (Lee et al., 2013). Mutations of NALCN in human cancer at a similar frequency of TP53, suggesting that NALCN could act as a tumor suppressor (Joerger and Fersht, 2016). Recently, Eric P. Rahrmann et al. found that trafficking of epithelial cells to distant tissues is regulated by NALCN, and loss of NALCN promotes cancer metastasis (Rahrmann et al., 2022).

However, no comprehensive pan-cancer analysis of NALCN has been conducted. There is still much to learn about NALCN’s role in cancer and whether it can be used as a diagnostic, prognostic or therapeutic biomarker. Thus, it is crucial to compare NALCN between different types of cancer, in order to understand how it differs and similarities through the pan-cancer analysis. In the present study, the role of NALCN in cancer was thoroughly examined and pan-cancer analysis was conducted through a comprehensive workflow. Our work showed that NALCN is aberrantly expressed and is highly associated with prognosis for most cancer types. NALCN is significantly related to clinicopathological features, immune-related biomarkers, immune-related genes and TIICs levels. The AUC show high diagnostic accuracy of NALCN in various cancers. Moreover, significant changes in methylation and genetic alteration of NALCN were found in multiple cancers. NALCN is enriched in multiple pathways involved in tumor development. Consequently, NALCN play a critical role in tumor immunity and prognosis, and possesses potential therapeutic and diagnostic implications.

NALCN were differentially expressed and related to poor prognosis in CRC patients. NALCN may bind with EMCN and promote the development of CRC (Huang et al., 2022). In this study, we found that NALCN was significantly differentially expressed in 16 types of cancer. NALCN protein levels have been found to be higher in most cancers based on IHC analysis. These findings suggest that NALCN could play an important role in the development of cancers and offers the prospect of advancing cancer diagnosis. A genome-wide association study showed that SNPs located in the genomic regions of NALCN have prognostic implication in advanced NSCLC (Lee et al., 2013). Circulating tumor cells (CTCs) and metastases are increased significantly through NALCN regulates malignant epithelial cells released into the blood from primary tumors (Rahrmann et al., 2022). Here, survival association analysis was conducted for each type of cancer using Cox regression analysis and KM survival curves, to examine the relationship between NALCN expression level and cancer prognosis, including OS, PFI, and DSS. According to the integrated results, we found that increased NALCN expression negatively impacted the prognosis of BLCA, CESC, COAD, HNSC, KIRC, KIRP, and SARC, but positively affected the prognosis of ACC and LGG. Additionally, NALCN expression was investigated in samples characterized by different clinicopathological features. The results showed NALCN expression was significantly correlated with tumor stage, histological tissue grades, molecular and immune subtypes. It is suggesting that NALCN is involved in the progression of various cancers with prognostic value. AUC of the ROC curve showed outstanding diagnostic performance of NALCN in various cancer types. NALCN proved high diagnostic value for 4 types of cancer and relative diagnostic value for 6 types of cancer. Consequently, NALCN may serve as a useful indicator of cancer occurrence and play a crucial role in assisting tumor diagnosis.

The mismatch repair pathway is important to maintain genome stability (Baretti and Le, 2018). Deficit of MMR is followed by MSI, leading to mutation accumulation in cancer-related genes and TMB aggravation (Yarchoan et al., 2017). TMB, MSI and MMR deficiency influence tumor initiation and were considered an independent predictor of treatment effectiveness with ICB (Sahin et al., 2019; Samstein et al., 2019; Zhao et al., 2019; Sha et al., 2020). Increasing studies showed that the prognosis of cancers was correlated with TMB, MSI and MMR (Germano et al., 2017; Stelloo et al., 2017; Yu et al., 2019). In our study, we examined the association between NALCN expression and TMB, MSI, and well-known MMR genes in different types of cancer. Most cancer types show strong correlations between NALCN expression and TMB, MSI, as well as MMR genes. It was indicating that NALCN may mediate tumorigenesis through genetic alterations and as a potential biomarker for predicting ICB responses. What’s more, we explored the relationship between NALCN expression and over 40 frequently occurring immune checkpoint genes. We found that there is a positive association between NALCN expression and many immune checkpoint genes. In addition, NALCN and chemokine genes or MHC-related genes exhibit a significant positive correlation. ESTIMATE score represents tumor purity and reflects both immune and stromal components. Cancer with low purity is considered to be in an advanced stage and has a poor prognosis (Yoshihara et al., 2013; Aran et al., 2015). In this study, NALCN expression is positively associated with ESTIMATE score in most tumor types, which indicate NALCN expression was related to tumor purity. It has been demonstrated that there is a clinical effect of TIICs on malignancy patients’ outcomes (Becht et al., 2016). Furthermore, immune infiltration analysis of NALCN was performed. We found that NALCN expression strongly positively relates the TIICs level. These findings demonstrate that NALCN highly involved in tumor immunity and plays a crucial role in tumor immune evasion.

DNA methylation is a novel predictor among the epigenetic mechanisms involved in tumorigenesis. Hong-qiang Chen et al. revealed the methylation level of NALCN is upregulated in the malignant transformation of human hepatocyte cell line (Chen et al., 2018). In the present work, we observed increased NALCN promoter methylation level and decreased NALCN mRNA expression appeared simultaneously across cancers. These results suggest that NALCN may mediate DNA methylation to regulate tumor progression. In addition to DNA methylation, downregulated of NALCN was also regulated by a genetic alteration (Chen et al., 2018). Fontanillo et al. found that the malignant state of glioblastomas was highly correlated with CNA of NALCN, with decreased expression level of NALCN (Fontanillo et al., 2012). NALCN mutations cause severe developmental and neurological disease (Bend et al., 2016; Fukai et al., 2016; Bramswig et al., 2018). Missense mutations generally result in gain-of-function phenotypes (Kschonsak et al., 2020). NALCN is affected predominantly by non-synonymous mutations which enriched in colorectal, gastric, lung, prostate, head, and neck cancers (Weinstein et al., 2013; Martincorena et al., 2017; Rahrmann et al., 2022). NALCN channel is closed by these cancer-associated mutations. Mutations in NALCN could facilitate cancer progression in the parallel and linear models (Klein, 2009). NALCN loss-of-function mutations could help to reveal some enigmatic characteristics of human cancer (Rahrmann et al., 2022). In this study, we found that NALCN mutation and CNA were found in most cancer types. Missense mutation and amplification were the major alteration type. The alteration event frequency of some cancer-related genes was significantly increased in the NALCN altered group, such as TP53, FGF14, SLC15A1. NALCN was also related to pathological stage and grade. These results show that NALCN could affect the malignant status and progression of cancer. In addition, the incidence of CRC, melanoma and esophagogastric adenocarcinoma is the highest top 3 cancers, which suggest that we should be concerned about the association between genetic mutations in NALCN and digestive system tumors.

Antimetastatic therapies have been difficult to develop due to the difficulty in identifying the primary tumor targets that drive metastasis (Ganesh and Massagué, 2021). NALCN deficiency resulted in abundant and persistent cell shedding, even without the primary tumor. Loss of NALCN promotes cancer metastasis. A dramatic increase in cancer metastasis with the deletion of NALCN in mice validates NALCN loss-of-function is a significant cancer metastasis driver (Rahrmann et al., 2022). Deleting NALCN from normal gastric stem cells, upregulation of epithelial-mesenchymal transition and invasion genes were observed within 72 h (Rahrmann et al., 2022). It is suggesting that NALCN regulate gene transcription which is similar to the reported calcium ion channel (Barbado et al., 2009; Wang et al., 2020). In this work, NALCN RNA and protein were aberrantly expressed and NALCN closely associated with clinicopathological features in multiple cancers, such as stage, grade, molecular and immune subtypes. The alteration event frequency of CCDC168, TP53, DOCK9, PCCA, FGF14, SLC15A1, ITGBL1, MYO16, NALCN-AS1, and FGF14-IT1 were significantly increased in the NALCN altered group. These results further verify the previous studies. However, more studies are needed to clarify the mechanisms that how NALCN regulates gene expression and cell shedding. Rahrmann EP et al. reveal that function manipulation of NALCN is a promising novel strategy to prevent cancer metastasis. Especially, drugs that can reopen the NALCN channel may be an effective antimetastatic therapy (Rahrmann et al., 2022). Therefore, in the future, targeted therapies can be developed for NALCN, and may enhance tumor treatment efficacy by combining with immunotherapy.

This study has some limitations need to be considered. Firstly, despite we gained some important insights about NALCN in tumors from bioinformatic analysis, our results need to be validated by additional biological experiments. Secondly, although NALCN expression in human malignant tumor was associated with immunity, as well as clinical survival, how NALCN affected clinical survival through the immune route still unsure. Thirdly, systematic bias exists given the multiple sources of information retrieved for the analysis. More studies are required to further investigate the role of NALCN in tumor and the potential therapeutic value of NALCN as an anticancer target.

In our first pan-cancer analysis of NALCN, we observed a significant differential expression of NALCN, association between NALCN and prognosis, clinicopathological features, diagnostic accuracy, immune-related biomarkers and genes, TIICs, DNA methylation, genetic alteration and tumor-related signaling pathways, which assist us understand NALCN’s role in tumorigenesis and metastasis. Taken together, our study revealed the vital involvement of NALCN in cancer and developed a framework for further study of NALCN in cancer. NALCN can be used as a prognostic biomarker for immune infiltration and clinical outcomes, and has potential diagnostic and therapeutic implications.

## Data Availability

Publicly available datasets were analyzed in this study. This data can be found here: The Cancer Genome Atlas (TCGA), https://www.cancer.gov/about-nci/organization/ccg/research/structural-genomics/tcga.

## Supplementary material

SUPPLEMENTARY TABLE S1 The number of colorectal cancer (CRC) and stomach adenocarcinoma (STAD) patients with D704H/Y missense mutation.

SUPPLEMENTARY TABLE S2 NALCN related gene list used for the enrichment analysis.
